# One-step synthesis of visible light CO_2_ reduction photocatalyst from carbon nanotubes encapsulating iodine molecules

**DOI:** 10.1038/s41598-021-89706-2

**Published:** 2021-05-12

**Authors:** Ayar Al-zubaidi, Kenta Kobayashi, Yosuke Ishii, Shinji Kawasaki

**Affiliations:** Department of Life Science and Applied Chemistry, Nagoya Institute of Technology, Gokiso-cho, Showa-ku, Nagoya, 466-8555 Japan

**Keywords:** Electrochemistry, Energy, Inorganic chemistry, Materials chemistry

## Abstract

We describe the synthesis and visible-light CO_2_ photoreduction catalytic properties of a three-component composite consisting of AgI, AgIO_3_, and single-walled carbon nanotubes (SWCNTs). The catalyst is synthesized by immersing SWCNTs encapsulating iodine molecules in AgNO_3_ aqueous solution, during which neutral iodine (I_2_) molecules encapsulated in SWCNTs transform disproportionately to I^5+^ (AgIO_3_) and I^−^ (AgI), as revealed from the characterization of the composite by Raman spectroscopy, X-ray diffraction, and X-ray photoelectron spectroscopy. In addition, photoirradiation experiments using a solar-simulator (AM1.5G) showed that the obtained three-component composite works as a CO_2_ photoreduction catalyst under visible light despite the wide band gap of AgIO_3_, suggesting possible transfer of the visible light-excited electron from AgI via SWCNTs.

## Introduction

The reduction of the CO_2_ levels in the air is an urgent necessity to solve climate problems such as the global warming^1–6^. In order for this reduction to be achieved sustainably, it should obviously not reply on artificial energy, and sustainable energy sources such as solar energy are required and being pursued by many researcher to achieve the task. Many researchers are trying to develop photocatalysts for solar light-induced CO_2_ electro-reduction^3,7–9^.

Among many candidates for the catalysts, AgIO_3_ has attracted considerable attention for multiple reasons^10,11^. AgIO_3_ has lone pair electrons from I^5+^ in the IO_3_^−^ anion, which can polarize it and contribute to forming a layered structure in crystals. The polarization and layered structure play significant roles in separating the photoexcited electrons and holes, which helps improve the catalytic activity. Furthermore, the bottom of the conduction bands mainly consists of I 5p and O 2p orbitals, whereas the top of the valence bands is occupied by Ag 4d and O 2p orbitals. The separate occupancy in conduction band (CB) and valence band (VB) by the orbitals from different groups is beneficial for the separation of photoinduced electrons and holes.

However, since the band gap of AgIO_3_ is 3.38 eV which is too wide for the carriers to be excited by visible light, the energy efficiency of AgIO_3_ as a solar light CO_2_ electro-reduction catalyst is very low^12^. Therefore, in order to improve the visible light-driven photoreduction efficiency, AgIO_3_ composites with other materials that can absorb visible light have been proposed and tested^13,14^. It has been reported in many publications that the photocatalytic properties of such composites are very promising. However, the synthesis processes of the composites are not very easy, and their fabrication cost should be a big problem for the practical application of these composites.

**Figure 1:**
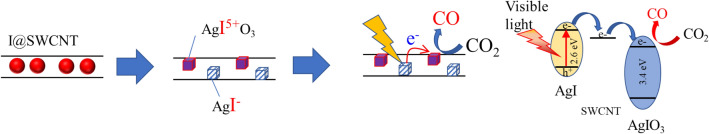
Schematic picture of the synthesis procedure of the three-component composite of AgI, AgIO_3_, and SWCNTs. The photocatalytic mechanism scheme of the composite under irradiation of visible light is also shown.

In the present paper, we describe a simple and highly scalable synthesis method of visible light CO_2_ photoreduction catalyst prepared as a three-component composite consisting of AgI, AgIO_3_, and single-walled carbon nanotubes (SWCNTs). Figure 1 shows a schematic picture of the electronic structures of the composite. The conceptual design of the catalyst involves the photoexcited electron of AgI being transferred via SWCNTs to AgIO_3_ to be used reduce CO_2_ to CO. Also and as shown in Fig. 1, the three-component composite itself is realized from iodine molecules encapsulated inside SWCNTs, and starts by iodine molecules receiving charge from the SWCNTs and converting into iodide ions that subsequently react to form AgI and AgIO_3_. By using iodine molecules encapsulated in SWCNTs, reaction products (AgI and AgIO_3_) should be prepared homogeneously in the SWCNT sample and the growth of the products should be restrained to be fine particles because of the regulated supply of iodine molecules.

In 1998, seven years after the discovery of carbon nanotubes, Smith reported a marriage between SWCNTs and C_60_ fullerenes, forming the material now called C_60_ peapods (C_60_@SWCNT)^15,16^. Transmission electron microscopy (TEM) images of the peapods, which showed well-ordered lineups of C_60_ molecules inside SWCNTs, were very impressive to many researchers. Some of the researchers have tried to synthesize other types of peapods encapsulating a variety of molecules. So far, fullerenes (C_60_, C_70_, metal-containing fullerenes), water molecule, organic molecules (carotene, TCNQ), inorganic molecules (sulfur, iodine, phosphorus) etc. have been encapsulated in SWCNTs^17–21^.

Many of the above-mentioned molecules are inserted into SWCNTs in a gas-phase process, in which the molecules should be sublimed and deposited in SWCNTs. However in 2013, our group reported another type of encapsulation, in which we inserted iodine molecules in SWCNTs by electro-oxidation of iodide ions^22^. This method was not only very easy and scalable, but also very effective, because we can control the amount of the encapsulated molecules just by changing the electrolysis time or electric current. We can insert iodine molecules by applying positive potential to the SWCNTs electrode in an electrolytic solution including iodide ions, then extract the inserted molecules just by changing the direction of the potential. The reversible insertion and extraction of iodine molecules can be used as battery electrode reactions^23,24^.

The investigation of the structural properties of the iodine molecules encapsulated in SWCNTs (I@SWCNT)have been investigated by many experiments such as TEM, X-ray absorption fine structure (XAFS) spectroscopy, and Raman spectroscopy^22–25^. These experiments revealed that the structure and electronic properties of the iodine molecules inserted by the electro-oxidation of iodide ions were identical to those of the molecules inserted by gas phase reaction. Iodine molecules were inserted in SWCNTs in the form of I_2_ regardless of the insertion method. We also observed that after the insertion, charge transfer from SWCNTs to iodine molecules causes some of the iodine molecules to convert to polyiodide ions (I_3_^−^, I_5_^−^ etc.). This was shown from the Raman spectrum of I@SWCNT, where characteristic Raman peaks of polyiodide ion were observed in the low wavenumber region. It is also known that this charge transfer reaction is strongly affected by temperature^25^.

Although the structural properties of the iodine molecules of I@SWCNT are well investigated, their chemical reaction properties are still not well understood. However, it is plausible that the iodine molecules should be reactive because they are energetically unstable in a restricted space. During our attempts to investigate the chemical reaction properties, we obtained a three-component composite consisting of AgI, AgIO_3_ and SWCNTs, in which I^5+^ ions in AgIO_3_ and I^−^ ions in AgI were simultaneously produced from I@SWCNT. In the present study, we describe this disproportionation reaction, and the structural and photocatalytic properties of the composite.

## Experimental

We purchased an SWCNTs sample from Meijo Nanocarbon Co., Ltd (EC type). We performed a purification acid treatment using to remove metallic impurities from the sample. The detailed purification procedure is described in our previous papers^22,24–26^. The removal of metal impurities was confirmed by thermogravimetric (TG) analysis. Iodine molecules were inserted into SWCNTs by electrochemical oxidation of iodide ions in electrolytic solution. To achieve electrochemical iodine insertion, we fabricated a three-electrode cell using paper-form SWCNTs as the working electrode, Pt as the counter electrode, and Ag/AgCl as the reference electrode. NaI aqueous solution (1 M) was used as the electrolyte, and we applied 1.8 V to the SWCNTs electrode for 15 min. After the encapsulation treatment, we washed the SWCNTs samples by distilled water to remove the iodine molecules deposited on the outer surfaces of SWCNTs, and dried the washed samples. The amount of the encapsulated iodine molecules was determined by TG measurements. We also performed Raman measurements to check the crystallinity. The nanostructure of the obtained samples was observed using a transmission electron microscope TEM (JEOL JEM-z2500) operated at 200 kV. The sample of SWCNTs encapsulating iodine molecules is denoted I@SWCNT.

To prepare the composite catalyst, we immersed the paper-form I@SWCNT sample in 20 mM AgNO_3_ aqueous solution for 10 h. The SWCNTs paper was then taken out and washed with distilled water. The washed SWCNTs sample was dried and characterized by TEM, SEM, XRD, XPS and Raman measurements. As will be discussed in the following section, the characterization revealed that the obtained sample is a composite consisting of AgI, AgIO_3_, and SWCNTs.

We investigated the CO_2_ photoreduction properties of the composite. The photocatalytic reduction of CO_2_ was carried out in an airtight cell using a solar simulator (XES-40S2-CE of SAN-EI ELECTRIC Co.) with power density of 100 mW/cm^2^ was used in a mode of AM1.5G. Prior to the irradiation, CO_2_ gas bubbling for 30 min was performed to presaturate the 0.5 M KOH aqueous solution with CO_2_. Gaseous samples from the airtight cell were taken by a syringe and manually injected into a gas-chromatograph (SHIMADZU GC-2014 equipped with methanizer and flame ionization detector) for analysis.

## Results and discussion

### Preparation and characterization of AgI-AgIO_3_-SWCNT composite

**Figure 2:**
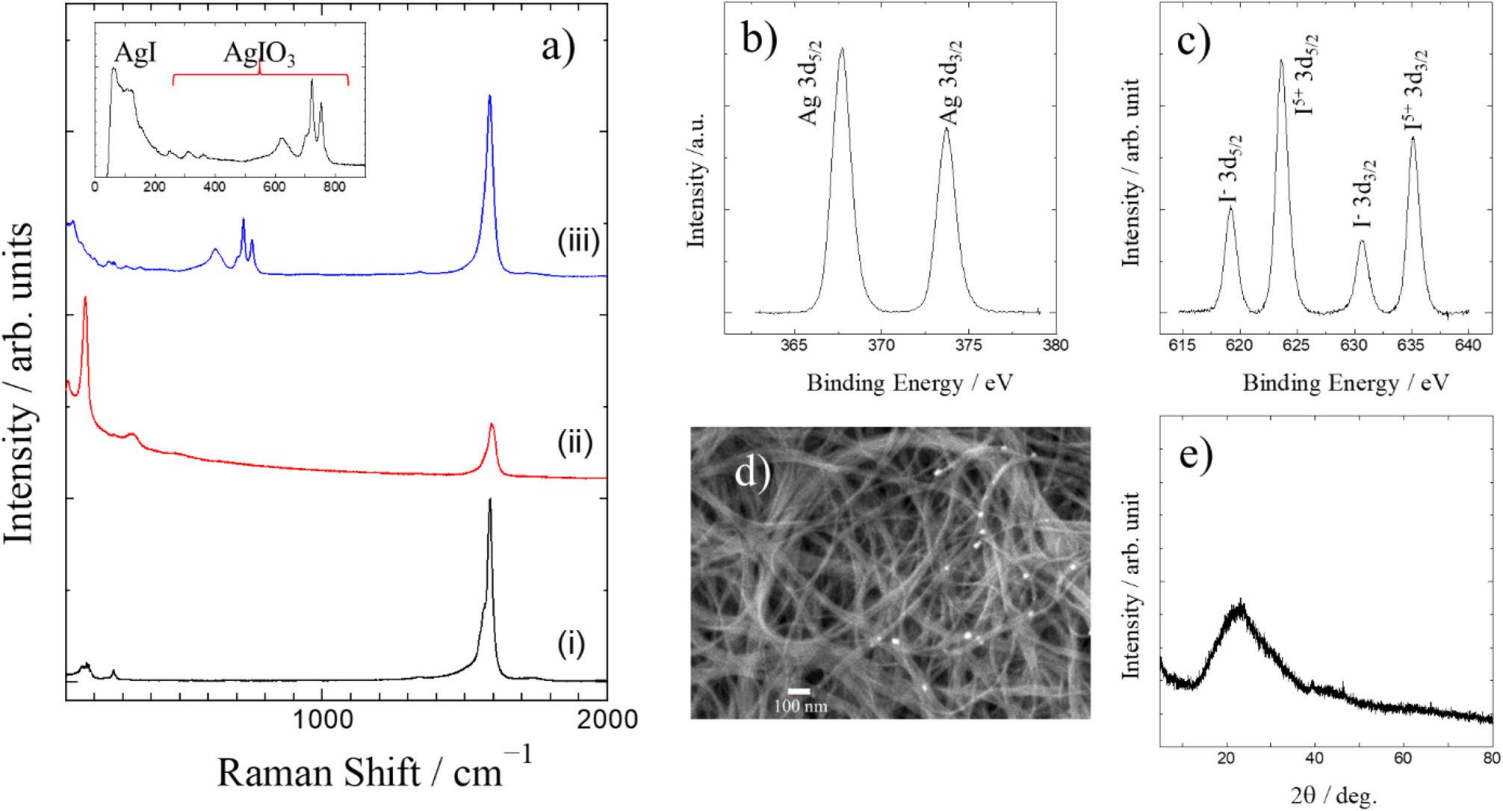
(**a**) Raman spectra of (i) pristine SWCNTs, (ii) I@SWCNT, (iii) AgI-AgIO_3_-SWCNT. The inset shows the low wavenumber region of the Raman spectrum of (iii). (**b**) Ag 3d binding energies and (**c**) I 3d binding energies on the XPS spectra of AgI-AgIO_3_-SWCNT. (**d**) SEM image of AgI-AgIO_3_-SWCNT. e) XRD pattern of AgI-AgIO_3_-SWCNT.

It was confirmed by TEM and SEM observations that the SWCNTs sample had low impurity content (Fig. S1a-c). Raman measurement also showed that the SWCNTs sample has good quality, because the intensity of D-band relative to G-band is quite weak (Fig. 2a-(i)). The peak positions of the radial breathing modes (RBM) observed in the low wavenumber region of the Raman spectrum can be used to determine the diameter distribution of the SWCNTs in the sample. However, it should be noted that only a part of SWCNTs having the gap energy of van Hove singularities close to Raman excitation laser energy in the sample mainly contributed to the observed Raman spectrum by resonance process. Therefore, in order to obtain a reliable diameter distribution by Raman measurements, we should measure the Raman spectra using different energy excitation lasers. Judging from Fig. 2a-(i), the SWCNTs sample has a variety diameter of SWCNTs ranging from 0.9 to 1.6 nm. TEM observation confirms this diameter distribution, while the main components have diameters of about 1.4–1.5 nm. After the encapsulation of iodine molecules, the Raman spectrum of the SWCNTs sample greatly changed as shown in Fig. 2a-(ii). Very strong peaks were observed in the low wavenumber region of the Raman spectrum of I@SWCNT. These peaks are characteristic of polyiodide ions. That does not indicate that iodide ions are adsorbed in SWCNTs. Iodide ions are oxidized to iodine molecules (I_2_), and those molecules are encapsulated in SWCNTs. However, after the encapsulation, charge transfer from SWCNTs to I_2_ molecules occurs and some of the I_2_ molecules are converted into polyiodide ions. In fact, even if we insert I_2_ molecules into SWCNTs by gas phase treatment, almost the same Raman spectrum having polyiodide ion peaks is observed (Fig. S2). As shown in Fig. 2a-(ii), the G-band peak position shifted toward a higher wavenumber upon the encapsulation of I_2_ molecules. The magnitude of the shift relates to the amount of the encapsulated I_2_ molecules. The iodine content in I@SWCNT was evaluated to be about 45% by TG measurement (Fig. S3).

Figure 2a-(iii) shows the Raman spectrum of the sample obtained by the immersion of I@SWCNT in AgNO_3_ aqueous solution. The polyiodide ion peaks of I@SWCNT are not observed in Fig. 2a-(iii). On the other hand, we can see the G-band of SWCNTs, which has almost the same peak position as the pristine sample, in addition to many peaks in the range100-800 cm^−1^. That indicates that iodine molecules encapsulated in SWCNTs reacted with AgNO_3_ aqueous solution. For convenience, the reacted SWCNT sample (Fig. 2a-(iii)) will be abbreviated as Ag-I-SWCNT. The inset of Fig. 2a shows the Raman peaks in the low wavenumber region of the spectrum of the reacted SWCNT sample. Compared to the reported Raman patterns of some iodine related compounds, the peaks in the range 150–800 cm^−1^ are found to be of AgIO_3_. The two strong Raman peaks of AgIO_3_ observed around 720 and 755 cm^−1^ can be assigned to the A1 and E modes of IO_3_^−^ ion. On the other hand, the peaks observed in the range 50–130 cm^−1^ are identical to those observed for AgI powder sample. Therefore, the observed Raman spectrum of Ag-I-SWCNT (Fig. 2a-(iii)) indicates that AgI and AgIO_3_ are simultaneously synthesized by the reaction of I@SWCNT and AgNO_3_ aqueous solution. The simultaneous formation of AgI and AgIO_3_ is supported by the following experiments.

Figure 2b shows the XPS spectra of Ag-I-SWCNT. The observed peak position of the Ag 3d_5/2_ binding energy is about 367.7 eV, which indicates positively charged Ag because the value is lower than the binding energy of Ag metal. On the other hand, and as shown in Fig. 2c, two kinds of iodine species exist in Ag-I-SWCNT. The two peaks at 623.7 and 635.2 eV can be assigned as I 3d_5/2_ and I 3d_3/2_ of I^5+^, while the peaks at 619.2 and 630.7 eV are attributed to I^−^. Therefore, the I 3d binding energies also indicate the simultaneous formation of AgI and AgIO_3_. Analyzing the XPS peak intensities of I^5+^ and I^−^, we calculated a content ratio of AgIO_3_ to AgI of about 2.2. One possible reaction for the simultaneous formation of AgI and AgIO_3_ is given by the equation:

$$6{\text{AgNO}}_{3} + 3{\text{I}}_{2} \to 2{\text{AgIO}}_{3} + 4{\text{AgI}} + 6{\text{NO}}_{2}$$

However, the content ratio for the reaction above should be 0.5. This discrepancy leads us to hypothesize that more complicated reactions (e.g. subsequent reaction of AgI) are involved in the synthesis process, although we currently do not have any evidence on the nature of such reactions.

If I_2_ molecules exist in the Ag-I-SWCNT sample, the I 3d_5/2_ peak of I- should have a shoulder peak or at least become broader, because I 3d_5/2_ of neutral I should be observed at around 620 eV, which is close to that of I^−^. However, the peak profile of I 3d_5/2_ peak of I^−^ is sharp and symmetrical. Therefore, I_2_ molecules do not exist in Ag-I-SWCNT sample, and it is plausible that all the iodine molecules reacted to AgI or AgIO_3_. This is consistent with the observation that polyiodide ion Raman peaks of I@SWCNT completely disappeared after the reaction with AgNO_3_ aqueous solution.

The SEM image of Ag-I-SWCNT sample (Fig. 2d) shows some deposited material on SWCNTs, but the deposits are not very clear. As mentioned above, the iodine content of I@SWCNT was about 45 wt.%. Assuming that all the iodine molecules reacted to form AgI and AgIO_3_, and that the deposits on SWCNTs consists of only these two crystals having the molar ratio of 1:2.2, the crystal volume of the deposition should be very small compared to SWCNTs volume. We performed XRD measurements to identify the deposits. As shown in Fig. 2e, we could not observe sharp diffractions, which means that the deposited materials should be aggregations of very fine crystals or amorphous materials. Amorphous AgI and AgIO_3_ are quite unlikely to be formed. In fact, in a different synthesis experiment, very weak diffraction peaks corresponding to the diffractions of AgI were observed (Fig. S4). Therefore, it is reasonable to assume that very fine crystals of AgI and AgIO_3_ are formed in the synthesis of Ag-I-SWCNT sample.

Up until now, we found that fine crystals of AgI and AgIO_3_ on SWCNTs can be prepared just by immersing I@SWCNT in AgNO_3_ aqueous solution. Although the detailed mechanism of the reaction is not yet clear, the disproportionation reaction from I^0^ to I^5+^ and I^−^ occurs very smoothly for iodine molecules encapsulated in SWCNTs.

### Photocatalytic properties of AgI-AgIO_3_-SWCNT composite

**Figure 3:**
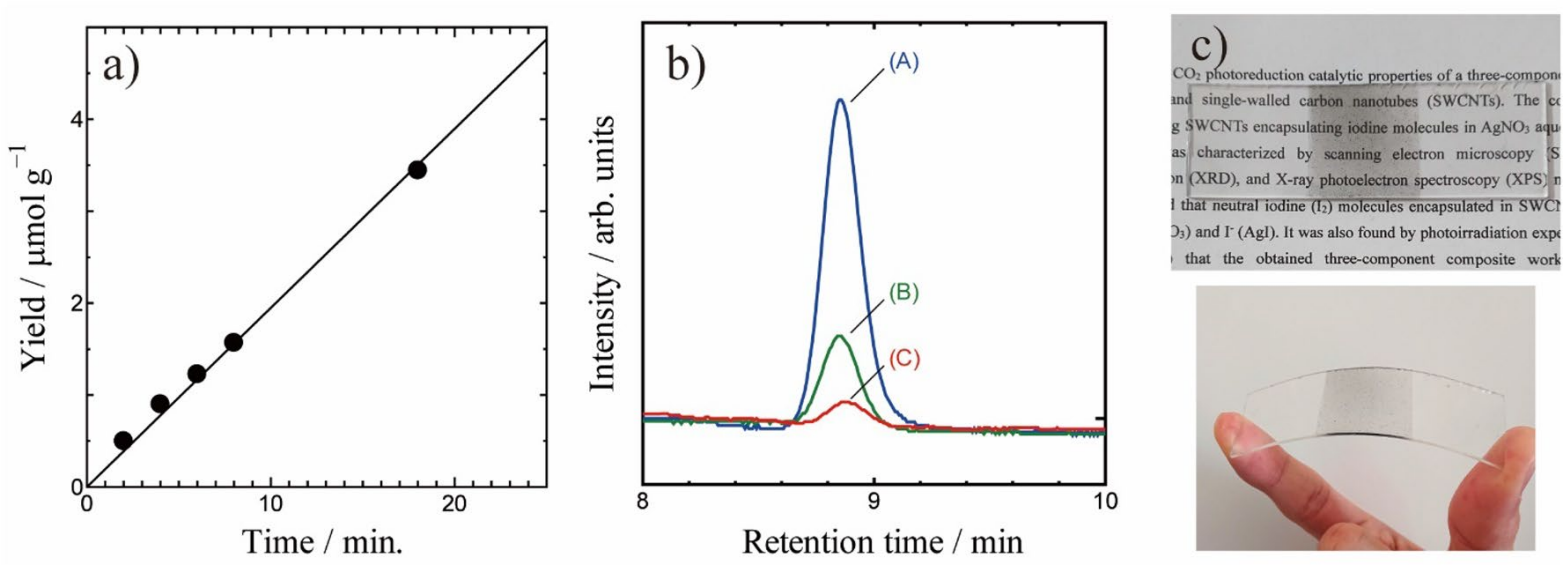
(**a**) Time dependence of CO yields over AgI-AgIO_3_-SWCNT under simulated solar light AM1.5G. (**b**) CO gas chromatography peaks of the AgI-AgIO_3_-SWCNT sample with (A) and without (B) UV-cut-filter. Data of pure AgI powder without UV-cut-filter (C) was also show as control experiment. (**c**) Transparent conductive film of AgI-AgIO_3_-SWCNT.

In this section, we describe the solar light CO_2_ reduction properties of AgI-AgIO_3_-SWCNT. The photocatalytic reduction of CO_2_ was carried out in an airtight cell containing CO_2_-saturated 0.5 M KHCO_3_ aqueous solution. Simulated solar light (AM1.5G, 100 mW/cm^2^) was irradiated for 2–20 h. After the photoirradiation, the gas in the airtight cell was analyzed by gas-chromatography. As shown in Fig. 3b, a peak that corresponds to CO was observed in the gas-chromatogram. On the other hand, CH_4_ was not detected by the chromatography. HCOOH is often produced by the reduction of CO_2_. However, in the present study, we could not detect HCOOH by NMR measurement of the aqueous solution of the airtight cell after the photoirradiation experiment. That does not mean that no HCOOH was produced by the CO_2_ reduction, because our NMR can detect HCOOH only in the case that the HCOOH concentration is more than 60 μmol/L. Since we could not see any CO peaks in the chromatogram for the blank test without the SWCNTs composite, we conclude that the composite works as a solar light CO_2_ reduction photocatalyst. Figure 3a shows the time dependent CO yields of this photocatalyst. As shown in Fig. 3a, the reduced amount is proportional with photo-irradiation time. The CO conversion efficiency is calculated to be 0.18 μmol/(g·h). We also investigated the chemical stability of AgI-AgIO_3_-SWCNT composite (Fig. S5). As shown in Fig. S5, the composite can work as photocatalyst at least up to 72 h, although the catalytic ability slightly decreased at 72 h.

As mentioned in the introduction section, we think that the photoexcited electron of AgI is transferred to AgIO_3_ via SWCNTs, and that the transferred electrons reduce CO_2_ to CO. In order to confirm that, we performed photoreduction experiments with a UV-cut filter (HOYA L42) that absorbs light with wavelengths less than 400 nm. In this condition, direct photoexcitation of AgIO_3_ is impossible because the band gap of AgIO_3_ is about 3.4 eV. Even in this case, the SWCNTs composite was able to reduce CO_2_ effectively (see Fig. 3b). On the other hand, in the case of only AgI sample (control experiment), only a limited amount of CO was detected (Fig. 3b). Namely, the electron transfer shown in Fig. 1 was confirmed. Furthermore, we also detected O_2_ generation when CO was detected by the photo-irradiation of the AgI-AgIO_3_-SWCNT composite (Fig. S6). It means that the reduction of CO_2_ to CO and oxidation of water to O_2_ occur in the photocatalytic process. The corresponding chemical reactions are summarized in Fig. S7. Similar electron transfer is already reported by Zheng for an Ag-AgI-AgIO_3_ system that also reduces CO_2_ to CO under visible light^11^. Zheng reported that the visible light-excited electrons of AgI are transferred to AgIO_3_ via Ag^11^. So, in their case, Ag worked as the electron transfer medium similarly to SWCNTs in our study. However, it is obvious that SWCNTs having fiber form are better as electron transfer medium because they effectively connect AgI and AgIO_3_.

Another merit of the SWCNTs composite is the easy fabrication of a flexible transparent conductive film of the CO_2_ photoreduction catalyst (Fig. 3c). The Transparent conductive film is easily prepared just by spray coating SWCNTs on a transparent polymer film or glass sheet. The obtained conductive film can be used as the electrode for subsequent iodine insertion, which is achieved easily by the electrooxidation encapsulation technique. After the encapsulation, we simply immersed the film in AgNO_3_ to prepare the three-component photocatalyst Ag-I-SWCNT as a transparent conductive film. This expands the scope of application of the CO_2_ photoreduction catalyst.

## Conclusion

We synthesized a three-component photocatalyst composed of AgI, AgIO_3_, and SWCNTs. The synthesis starts by preparing iodine-encapsulating SWCNTs (I@SWCNT) sample with about 45 wt.% iodine through applying 1.8 V (vs. SHE) to paper-form empty SWCNTs electrode in 1.0 M NaI aqueous solution for 15 min. We then obtained the three-component composite of AgI, AgIO_3_, and SWCNTs (AgI-AgIO_3_-SWCNT) by simply immersing I@SWCNT in AgNO_3_ aqueous solution. The obtained composite was characterized by Raman measurements, revealing that AgIO_3_ exists in the composite, because a characteristic Raman peak pattern of AgIO_3_ was observed in the spectrum of the composite. The Raman spectrum also indicated the existence of AgI in the composite, which was also confirmed by observing weak diffraction peaks of AgI in the XRD pattern and I 3d binding energies in the XPS spectra of the composite, all clearly indicating the coexistence of I^5+^ and I^−^. These measurements revealed that neutral iodine (I_2_) molecules encapsulated in SWCNTs transform disproportionately to I^5+^ (AgIO_3_) and I^−^ (AgI). We found that the obtained three-component composite works as a CO_2_ photoreduction catalyst under visible light, overcoming the issue of the otherwise wide bandgap of AgIO_3_, suggesting that the visible light-excited electron of AgI is transferred to AgIO_3_ via SWCNTs, and that the transferred electrons reduce CO_2_ to CO.

## Supplementary Information

Supplementary Figures.
