# Supplementary material for: One-step synthesis of visible light CO2 reduction photocatalyst from carbon nanotubes encapsulating iodine molecules
Source: Sci Rep. 2021 May 12;11:10140. doi: 10.1038/s41598-021-89706-2 (PMC8115251; doi:10.1038/s41598-021-89706-2)
Supplement: Supplementary file 1 — Supplementary Figures. [file 41598_2021_89706_MOESM1_ESM.pdf]

Supporting Information

One-step synthesis of visible light CO_2 reduction photocatalyst from carbon nanotubes encapsulating iodine molecules

Ayar Al-zubaidi[1], *Kenta Kobayashi*[1], *Yosuke Ishii*[1,*], *Shinji Kawasaki*[1,*]

[1]Department of Life Science and Applied Chemistry, Nagoya Institute of Technology, Gokiso-cho, Showa-ku, Nagoya 466-8555, Japan.

Corresponding Author

* E-mail: ishii.yosuke@nitech.ac.jp, kawasaki.shinji@nitech.ac.jp.

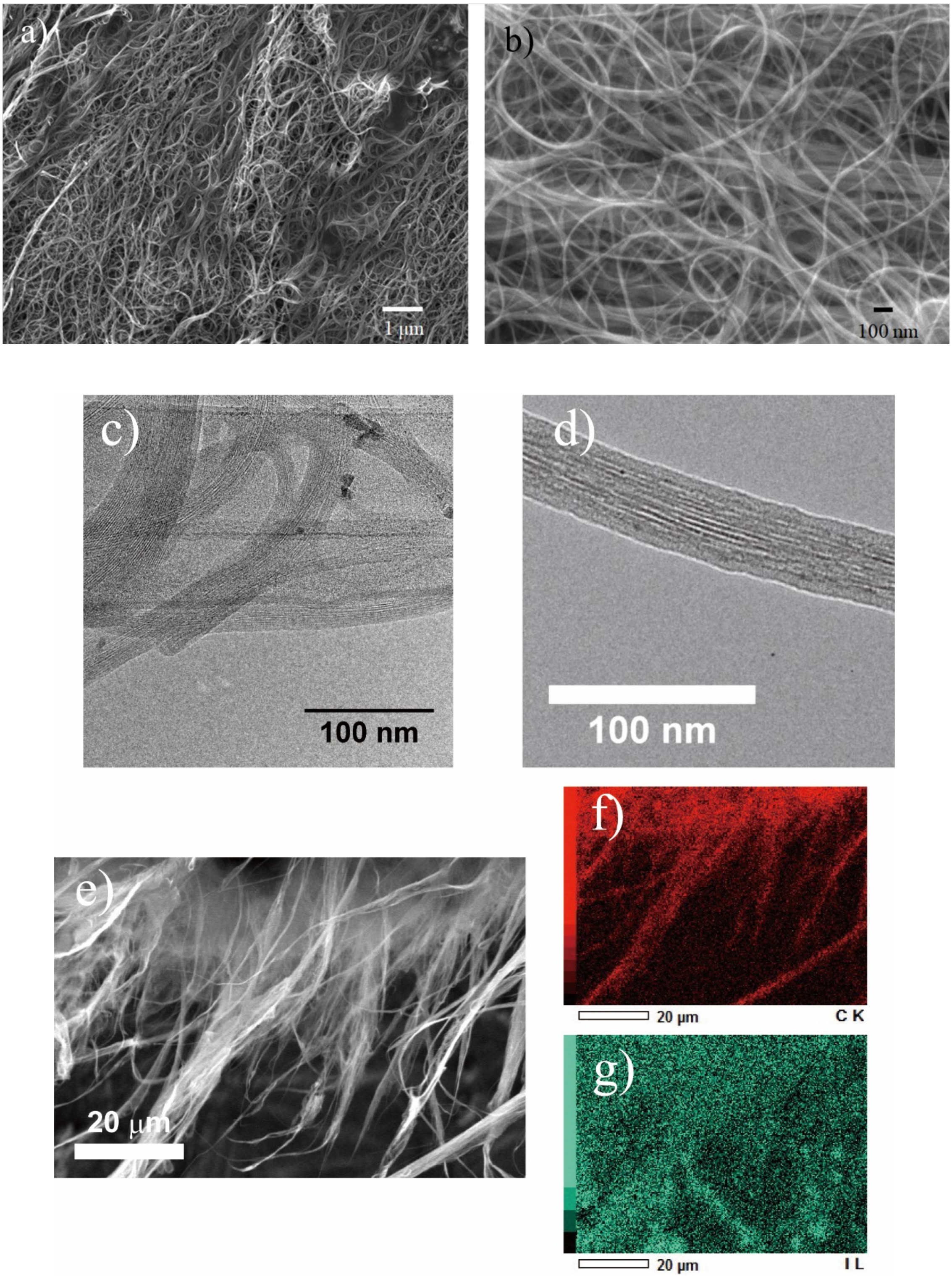

Fig. S1 (a, b) SEM and (c) TEM images of pristine SWCNT. (d) TEM and (e-g) SEM-EDS images of I@SWCNT.

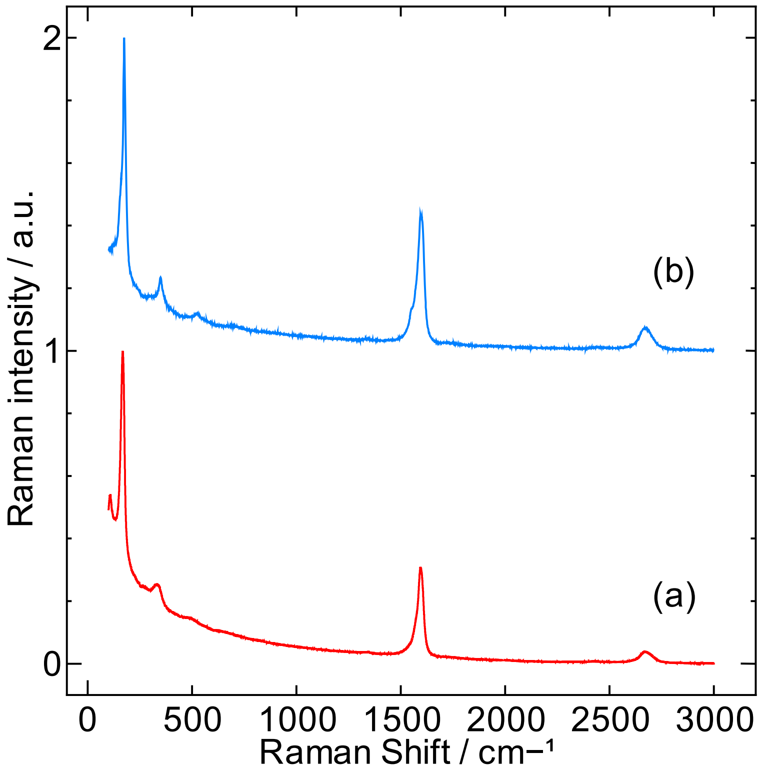

Fig. S2 Raman spectra of I@SWCNT prepared by (a) electro-oxidation method and (b) gas-phase method.

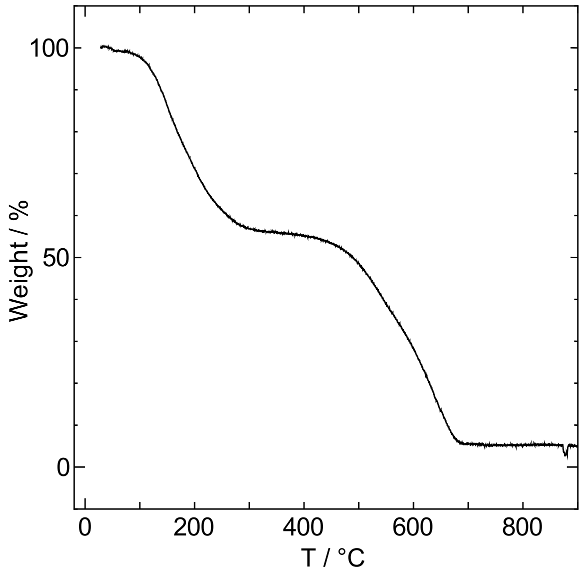

Fig. S3 Thermogravimetric curve of I@SWCNT.

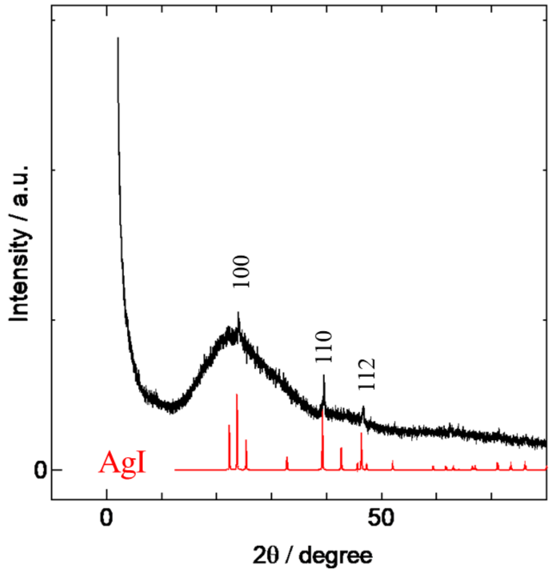

Fig. S4 Observed XRD pattern (black) of Ag-I-SWCNT sample obtained by another experimental run different from main text and JCPDS pattern of AgI.

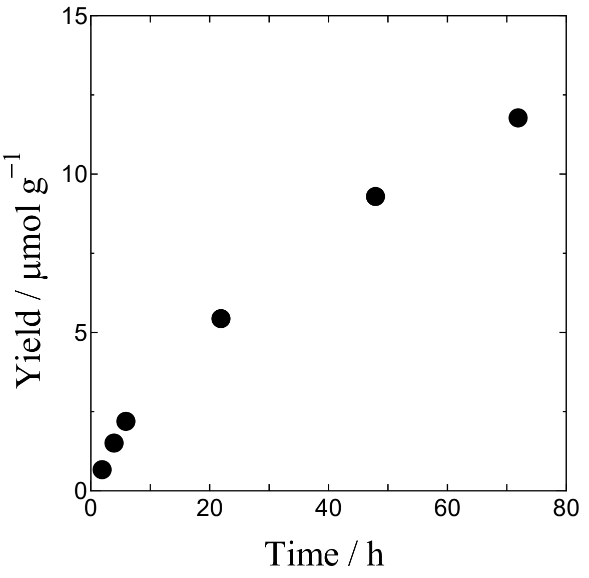

Fig. S5 Time dependence of CO yields over the AgI-$AgIO_3$-SWCNT sample under AM1.5G.

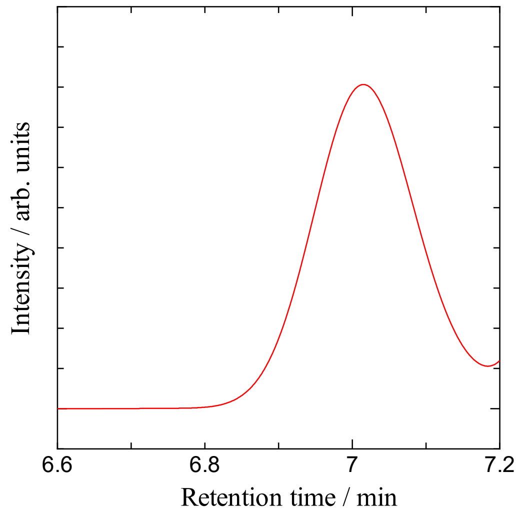

Fig. S6 O_2 gas chromatography peak of the AgI-$AgIO_3$-SWCNT after photo-irradiation.

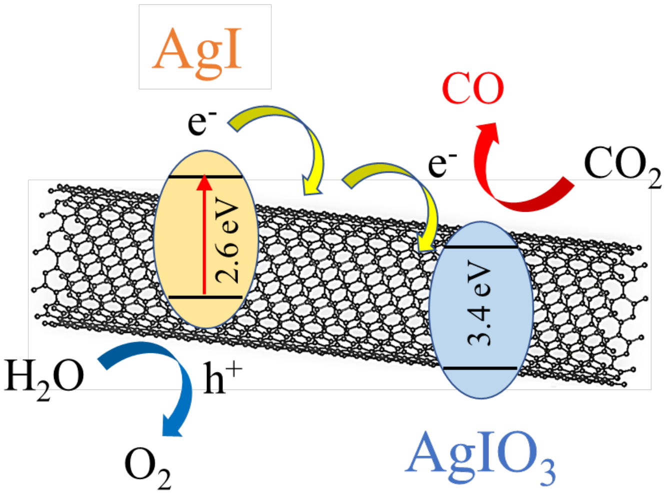

$$2CO_2 + 4H^+ + 4e^- \rightarrow 2CO + 2H_2O$$

$$2H_2O \rightarrow O_2 + 4H^+ + 4e^-$$

Fig. S7 Reaction scheme of the AgI-AgIO$_3$-SWCNT.
